# Shared structure of fundamental human experience revealed by polysemy network of basic vocabularies across languages

**DOI:** 10.1038/s41598-024-56571-8

**Published:** 2024-03-11

**Authors:** Yuzhu Liang, Ke Xu, Qibin Ran

**Affiliations:** 1 School of Liberal Arts, Nankai University, Tianjin, 300071 China; 2 Tianjin Social Science Laboratary, Nankai University, Tianjin, 300071 China

**Keywords:** Neuroscience, Psychology

## Abstract

How are concepts related to fundamental human experiences organized within the human mind? Our insights are drawn from a semantic network created using the Cross-Linguistic Database of Polysemous Basic Vocabulary, which focuses on a broad range of senses extracted from dictionary entries. The database covers 60 basic vocabularies in 61 languages, providing 11,841 senses from 3736 entries, revealing cross-linguistic semantic connections through automatically generated weighted semantic maps. The network comprises 2941 nodes connected by 3573 edges. The nodes representing body parts, motions, and features closely related to human experience occupy wide fields or serve as crucial bridges across semantic domains in the network. The polysemous network of basic vocabularies across languages represents a shared cognitive network of fundamental human experiences, as these semantic connections should be conceived as generally independent of any specific language and are driven by universal characteristics of the real world as perceived by the human mind. The database holds the potential to contribute to research aimed at unraveling the nature of cognitive proximity.

## Introduction

Concepts correspond to our understanding of regularities in the world, which are generalizations about the kinds of things that exist and the properties that they have^1^. In a universalist view, common-sense notions map onto conceptual categories that we acquire independently of any language experience. This indicates that concepts, especially common concepts (e.g., SUN, WATER, HUMAN) that denote fundamental human experiences, are universal to human cognition. These common concepts are also part of the basic vocabulary across languages. This universality is due, in part, to the fact that the relevant linguistic categories and structures are straightforward mappings from a preexisting conceptual space that is programmed into our biological nature^2–4^.

However, human language relies on a finite lexicon to express an infinite set of ideas. To express new ideas, we can extend existing words beyond their original meanings rather than inventing new words. This extension of existing words to new meanings creates polysemy, where a single word form expresses multiple related senses^3^. The extended meanings of polysemous words are sometimes related in a seemingly arbitrary and opaque way, and they may reflect distinct features of cultural, historical, and environmental backgrounds, in addition to properties of human cognition. There is considerable cross-linguistic variation in the sets of meanings of the same polysemous word^5^. Even a seemingly simple concept such as WATER encompasses rich information about what it refers to, including related ideas such as “river”, “rain”, “soup”, “tear”, “sweat”, “perfume” and so on. However, these meanings are not shared across languages. According to Zipf^6^, the number of meanings of a word is positively correlated with its frequency of use. Therefore, the words that express these fundamental concepts are most likely to have multiple meanings.

Despite the acknowledgement of a semantic structure inherent to our nature as proposed by Chomsky^4^, the path of polysemic extension for a common concept remains uncertain due to significant cross-linguistic variations. We have chosen to collect core concepts related to fundamental human experiences and their corresponding meanings as expressed through polysemous words. This allows us to observe and explore the diverse extension paths of these fundamental concepts. Moreover, polysemy is the result of semantic changes. Both universal and language-specific semantic shifts can be considered a window onto human cognitive mechanisms^7^. Polysemy is essentially cognitive phenomenon and thus reveals general cognitive processes^8^. Analyzing how polysemous words are extended across different languages may provide insights into how concepts related to fundamental human experiences are organized within human cognition.

### Current dataset and database resources for studying polysemy in cross-linguistic basic vocabulary

Previous studies on cross-linguistic polysemy have typically focused on a single semantic domain, limiting our understanding of cross-domain polysemy patterns. While questions about how languages distribute meanings across vocabularies have a long-standing tradition in linguistics and philosophy, global-scale studies have been restricted to domains such as body parts^9^, physical entities^10^, perception verbs^11^, time-related words^12^, and emotion words^13^. Although these studies examine universal and core semantic domains, they fail to capture the full range of human experience, which spans multiple domains that are often interconnected. In fact, the semantic representations in the human brain are adjacent, suggesting that fundamental semantic domains can be connected or overlapped^14,15^. Therefore, there is a need for broader studies that explore cross-domain polysemy patterns on a global scale.

Other studies about polysemy fell into such a dilemma that they were conducted on members of western, educated, industrial societies. For example, Srinivasan and Rabagliati provided evidence of some cross-linguistic variation with respect to patterns of polysemy, most patterns that were present in English were also generally present across languages^5^. These findings of polysemy, which were observed across languages, may have been influenced by Anglo-centrism. And some of concepts they assessed, such as NEWSPAPER, BOTTLE, BEAM, RABBIT, are not common in all languages. Cultural and technological differences between speakers of different communities may cause some variation. This raises the possibility that polysemy in other languages has developed due to contact with English, resulting broad similarity.

Currently, the available databases do not provide sufficient sample sizes of the polysemy resources we are interested in studying. While multiple databases serve as the basis for studying synchronic polysemy, the “Database of Semantic Shifts” by Zalizniak et al. focuses primarily on considerably frequent, prominent, and significant semantic correlations^7^. For instance, the meaning of “hand” can shift to include meanings such as “move (board games)”, “direction”, “fascicle (cluster of flowers or berries)”, and “the day after tomorrow”. However, this approach may overlook idiosyncratic relations that are less common but still important for polysemy extension, such as the meanings of “hand” that include “control”, “method”, and “power”. In Arabic, Czech, Hausa, Persian, Portuguese, Turkish, and Urdu, “control” serves as an extended meaning of HAND, and in Chinese, Lao, Persian, Thai, Turkish, Urdu, and Vietnamese, “method” also serves as an extended meaning of HAND. Additionally, in Hebrew, Lao, Latin, Urdu, and Persian, “power” is an extended meaning of HAND. Notably, CONTROL, METHOD, and POWER are not linked to HAND in the Cross-Linguistic Colexifications (CLICS) database^16,17^. CLICS identifies cross-linguistic polysemes by collecting colexifications, multiple concepts expressed with the same word form, across multiple languages. However, despite its inclusion of 2919 concepts, the database overlooks certain associations between these concepts. These connected concepts often serve as the base and extended meanings, respectively, within a single word.

### Polysemy of basic vocabulary: a dataset collected from medium-sized dictionaries in 61 languages

In this paper, we present an open-source database of Cross-Linguistic polysemes for basic vocabularies aimed at collecting common concepts and exploring their extended meanings as comprehensively as possible. Our database comprises the senses of 60 basic vocabularies across 12 semantic domains from 61 language varieties spanning 13 language families, totaling 11,841 senses of 3736 entries. This paper delves into some classic concrete concept categories, such as physical entities, human entities, and body parts, as well as characteristic categories like qualities, quantity, physical attributes, and other common or frequent domains, including motion verbs, pronouns, and numerals. We manually collected data from medium-sized dictionaries as they contain sufficient entries and meanings for our purposes. This method enables us to gather intricate and multifaceted meanings while avoiding including homonymous words in the database.

Researchers are increasingly using graphic structures and spatial representations that demonstrate distance for visualizing semantic databases^18–20^. Croft has conducted a typological study of word polysemy to construct a quantified network of semantic similarity among basic vocabulary items for comparative historical research^21^. We aim to uncover cross-linguistic regularities and variations in polysemous words within our database. To achieve this goal, we have created semantic maps that describe the polysemy patterns in a synchronic typological sample^21^. Our semantic maps aim to provide a clear network diagram that integrates intricate and complex semantic extension relations, allowing us to establish the semantic structure of these words.

This paper is structured as follows. In section “Cross-linguistic database of polysemous basic vocabulary”, we describe how we built our open-source resource, the cross-linguistic database of polysemous basic vocabulary. In section “Overview of basic findings in the database”, we provide an overview of the basic results obtained from the database. In section “Polysemous semantic networks of basic vocabulary across languages”, we present two subsections to illustrate the research potential of the database. Specifically, we first use automatically plotted weighted semantic maps to reveal cross-linguistically shared semantic structures. We then show the main findings from the established semantic maps in section “Polysemous semantic networks of basic vocabulary across languages”. Finally, section “Conclusion” concludes the paper.

## Cross-linguistic database of polysemous basic vocabulary

We examined 60 basic words (see Table 1 across 61 languages). Basic or core vocabulary is universal and relatively culture-free, and thus is less subject to replacement than other kinds of vocabulary. These are not actually lists of “words” per se, but rather of concepts for which relevant words with the corresponding meanings are sought in the languages investigated^22^. “Concepts” exist independently of language and correspond to our understanding of regularities in the world, which are generalizations about the types of things that exist and their properties^1^. All words representing “concept” are now capitalized in the main text. Our concept list was drawn from the Swadesh basic vocabulary lists, encompassing physical entities, body parts, animals, and other categories.

**Table 1:** Concepts and their corresponding semantic domain.

Semantic domain	Concept
Natural entities	WATER, FIRE, STONE, EARTH/SOIL, RAIN, SKY, SUN, MOON, NIGHT, TREE, BIRD, DOG
Human entities	HUMAN, PERSON, MAN, WOMAN
body parts	HEAD, EYE, EAR, MOUTH, HAND, FOOT, HEART, BONE, BLOOD
Bodily motions	EAT, DRINK, SAY, SEE/LOOK, HEAR/LISTEN, STAND, SLEEP, GIVE, GO, COME, DIE
Quality	GOOD, BAD
Quantity	MANY
Physical attributes	SQUARE, ROUND, BLACK, WHITE, BIG, LONG, NEW, OLD
Personal pronouns	I, YOU
Demonstrative	THIS, THAT
Interrogative	WHO, WHAT
Numerals	ONE, TWO, THREE
Negation	NOT

The criteria for determining basic concepts are based on the Swadesh 100 list, with two key standards. First, concepts are included if they are more universally present in world languages. For instance, in the Swadesh's 100 core word list, FISH and MOUNTAIN are present. However, since fish may not be common in arid regions and mountains may not be prevalent in plains, such cases are excluded from the basic concept list. Some vocabularies were also excluded from our list due to the difficulty of finding direct counterparts in certain languages; for example, GREASE was often narrowed down to a more specific concept, such as animal fat, or even replaced by another concept, such as oil^23^. Secondly, the basic concept list excludes concepts that appear later in implicational hierarchies. For example, in the category of color words, there are numerous terms like BLACK, WHITE, RED, YELLOW, and GREEN. If a language has distinct terms for RED, YELLOW, and GREEN, it is implied that it also has terms for BLACK and WHITE^24,25^. The basic concept list focuses on retaining only the most fundamental concepts, such as BLACK and WHITE. The final 60 concepts can be grouped into 12 distinct semantic domains. Although NEW and OLD may not be strictly categorized as physical attributes, they are associated with such attributes in certain contexts.

The selection criteria for languages consider both phylogenetic and geographic diversity. We prioritized languages with accessible, high-quality dictionaries, including monolingual and bilingual ones with semantic metalanguages (English and Chinese). Due to uneven dictionary resources across languages, our selection aimed for phylogenetic and geographic diversity rather than a perfect balance. In the end, the 61 languages represent 13 language families, including Indo-European and Austronesian, and feature isolated languages like Basque and Korean. Using these resources, we identified word forms expressing the selected concepts, documenting both the basic meanings and additional meanings listed in the dictionaries. If the dictionary is in Chinese, we provided concise English translations for the meanings.

Our database was initially intended to contain only 3660 dictionary entries (61 languages × 60 concepts), with each concept corresponding to a dictionary entry in a specific language. However, the actual number of collected entries exceeded 3660, reaching 3736. This discrepancy arises from two main situations. Firstly, a single concept may not always be associated with only one dictionary entry, as there are instances of synonyms or near-synonyms. Secondly, for the concept EARTH/SOIL, while it represents a single concept, it is not colexified in many languages, resulting in two dictionary entries. A similar situation exists for concepts like SEE/LOOK and HEAR/LISTEN. The concepts of SEE (Chinese: jiàn) and LOOK (Chinese: kàn) are often colexified in many languages, as are HEAR and LISTEN^11,26^. It means that they are often expressed by the same word or form. For instance, the colexification of SEE and LOOK is expressed as “görmek” in Turkish. In semantic metalanguages and certain languages, multiple word-forms may be used to express the same concept. Although neither LOOK nor LISTEN is included in the Swadesh list, the dictionary entries for these concepts were still identified in the selected languages. As SEE and LOOK are viewed as the same concept, they were combined into a single node when constructing the semantic map. Similarly, HEAR and LISTEN were also combined into a single node.

Criteria for extracting meanings from dictionary entries. (1) Avoid including meanings from a homophone that has the same word form corresponding to the target concept. (2) Avoid including meanings whose understanding requires a specific ethnic cultural background. For example, in Chinese, “fire” might refer to a specific health condition, and “eye” might indicate the weak beats in traditional Chinese opera rhythms. These meanings would not be included. (3) Avoid including meanings that only appear in specific structures. For example: “so long as.” (4) Avoid including meanings referring to recent technological terms. Retain the following meaning distinctions: Changes in the subject and object for verbs; changes in the referred entity for nouns; changes in the subject described or in the degree for adjectives and adverbs.

To avoid confusion, clear definitions for the terms used in this study are provided. We employ words to convey concepts. In turn, words connect abstract knowledge with the tangible forms of sounds, letters, or signs in a particular language. The interpretations or various meanings of this word, found in the dictionary entries of this language, are referred to as “meaning.” This “meaning”, distinct from the “sense” mentioned later, is raw and not simplified, remaining language-specific. When cross-linguistically comparing the meanings found in dictionary entries, the individual or multiple meanings of a monosemous or polysemous word are represented as simpler semantic units known as “senses,” following a functionally-based, language-independent criterion^27^. Particularly when establishing a semantic network, these meanings are termed “senses.” “Senses” form elements of a polysemous network. Choosing a specific sense serves as the pivot for constructing a universal lexical map, preventing the risk of starting an open-ended map with ever-shifting boundaries. Simultaneously, it's an arbitrary choice, as any sense can serve as a starting point before drawing any lexical map^27^. In the polysemous semantic network, 60 concepts serve as the equivalent of specific senses, functioning as the pivot of the network. This is because our focus is solely on these 60 fundamental concepts. All words representing a “sense” are denoted in the text within angled brackets “< >”.

We have made the final version of the dataset available in a CSV format, which includes the following fields:

- Language name (e.g., Arabic),
- Language family (e.g., Afro-Asiatic),
- Concept (e.g., HAND),
- Word written in the corresponding language (e.g., يد, which denotes HAND in Arabic),
- Number of senses (in this case, there are 12 senses of HAND in Arabic),
- Synonyms number (Synonyms number is marked as “0” if no other word expresses the same concept, or as the number of synonyms if applicable. For example, in Burmese, there are two words expressing the same concept, both meaning HEAR/LISTEN, so their synonyms numbers are both marked as “2”.)
- Senses: 12 senses of HAND in Arabic. They are <hand>, <arm>, <be involved in>, <control>, <eat>, <engage in>, <good at>, <handle(v)>, <in front of>, <lifetime>, <generations>, <palm>, <propose>.

Finally, the database offers 11,841 senses from 3736 dictionaries entries. On average, individual forms in the sample express 3.16 senses, indicating that cases of lexical items with two or three extensive (or additional) senses (excluding base sense) are frequent. For instance, the word denoting EYE in Arabic has the largest number of senses, with 21 in total.

## Overview of basic findings in the database

The dataset provides insight into the rich extension of meanings for basic vocabulary words. The senses of common concepts demonstrate wide diversity across languages. In our analysis, we not only investigated the senses that are commonly shared across different languages but also explored the senses that are rare or uncommon. One can argue that there are no natural constraints on the possible directions and outcomes of semantic change. With sufficient creativity and boldness, it is conceivable to assert a semantic connection between nearly any two words in existence, according to Hock^28^. Physical entities have diverse properties, resulting in complex and varied extensional senses that cannot be fully generalized, as in the case of WATER. The concept WATER has multiple senses, including <liquid>, <fluid>, <waters>, <river>, <lake>, <sea>, <rain>, <flood>, <alcohol>, <juice>, <beverage>, <soup>. They are several cases where the basic sense leads to a specialized sense <rain>, <flood>, <alcohol>, <juice>, <beverage>, <soup>, wider sense <liquid>, <fluid>, and larger sense <river>, <lake>, <sea>. These senses that were more semantically related to base senses are shared across languages. Some senses are not shared across languages. The concept WATER also includes senses denoting body fluid, such as <urine>, <tear>, <sweat>, <saliva>, <secretion>, <semen>, <rheum>, <blood>, which are found in Germanic and Romance languages. There are other examples of culturally unique senses: <stupor> (Spanish), <aquatic>, <water-based>, <wash>, <baths>, <leak> (Italian), <perfume> (German), <lotion> (Czech), and <suck> (Māori). There are some senses that are related in seemingly arbitrary and opaque ways. For example: <slope of a roof> (Spanish), <brilliance shown by gems>, <self-respect> (Arabic), <radiance on the face>, <cloud>, <lotus>, <tip of an elephant's trunk> (Bengali), <delicate>, <sharp>, <soft>, <fresh>, <gross> (Farsi), <nonsense, color, interest> (Hausa), <draw in the breath audibly> (Turkish), <edge with a lace>, <drowsy>, <bad humors in the body> (Telugu). Even the connection between a word's basic and extended meanings can be understood in various ways.

Moreover, the level of cognitive association between the basic and additional senses of physical entities is highly susceptible to external geographic factors. This can lead to difficulties in understanding the relationship between certain senses for speakers of different languages, and can also highlight cultural disparities. For example, in Mongolian, EARTH/SOIL also means <epidermis>, <turf>, and <complexion>. In Chinese, it can also refer to <opium>, while in Turkish, it means <heroin>. In Indonesian means <mess> in Hindi. STONE can be extended to <kernel> in English, German, and Māori, <grain> in Finnish, and <seed> in Norwegian. It can also refer to <core of a boil or abscess> in Māori, and <pupil of the eye> in Norwegian. It can also mean <cheat> and <drive a wedge between> in Mongolian, and <blockhead> in Latin. In Lao, RAIN can also refer to <season>, while in Thai and Lao, it can mean <year>. SUN can refer to <sunflower> in Malay, French, Greek, and Arabic, <the sun-plant> in Bengali, and <sunfish> in Arabic.

**Figure 1:**
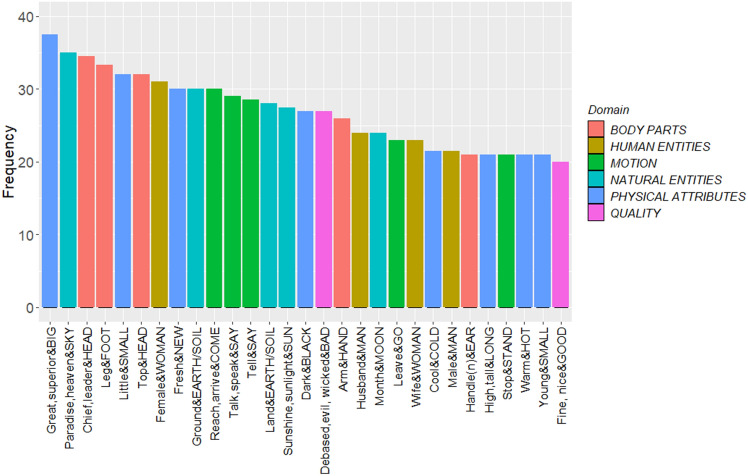
The most frequent extended senses across languages. The label on the x-axis represents the most frequent extended senses and their corresponding concepts.

However, others have suggested that more commonly related senses across languages are those that require less cognitive effort to relate^26^. The most-commonly shared senses across different languages (see Fig. 1) demonstrate the universality of human cognition. For example, FOOT can refer to the <leg> or <the palm of the foot> in 32 languages including Basque and Vietnamese. HEAD means <the top part> in 18 languages. HEART can be referred to as the <center> in 13 languages. The high-frequency senses are more logically and understandably connected with base senses.

We examined the number of senses associated with various concepts, identifying those with the most and least senses. Certain concepts exhibit a higher likelihood of being polysemous than others. The following are the top 10 concepts with the largest number of senses: GO (5.72), GOOD (5.62), GIVE (5.39), HEAD (5.36), STAND (4.84), BIG (4.78), SEE/LOOK (4.68), BAD (4.64), EAT (4.55), COME (4.34). On average, the concept GO is associated with 5.72 senses. Additionally, we found 10 concepts with the fewest senses: THIS (1.97), DOG (1.93), THAT (1.93), BIRD (1.87), TWO (1.81), NIGHT (1.75), RAIN (1.63), I (1.41), THREE (1.36), YOU (1.33). The numbers in brackets represent the count of senses expressed by the single form of each concept. Among common concepts, words denoting GO, GOOD, GIVE, and HEAD rank as the most frequent in the mental lexicon, a phenomenon attributed to the increase in word definitions with word frequency^29^.

Based on the word class of concepts in the metalanguage, we conducted a statistical analysis of the number of senses connected to each concept but found no meaningful patterns or consistent rules. On average, verbs express 3.17 senses which are the most. The number of adjective senses (2.45) and the number of noun senses (2.20) rank second and third respectively. The quantitative difference in senses between nouns and verbs may help explain why there are differences in the ability of distributional models to acquire noun and verb semantics^30^. Gentner showed that children find verb concepts harder to learn than noun concepts^31^, and Markman and Wisniewski present evidence that different cognitive operations are employed when comparing two nouns or two verbs^32^. However, numerals in Norwegian, Swahili, and Armenian possess more senses than nouns, while adverbs in Lao, Lingao, and Japanese have more senses than other parts of speech. These numbers of senses are randomly distributed to some extent and do not follow consistent patterns due to the various syntactic constraints of each language^5^. Consequently, it is challenging for grouping descriptive statistics to fully capture the regularities of polysemy from the complex semantic relationships. It is necessary to construct a semantic map that can provide a macroscopic view of the complex semantic extension pathways and reveal the regularities that may not be evident from general statistical data.

While the Cross-Linguistic Database of Polysemous Basic Vocabulary has not undergone a thorough comparison with CLICS, we compared several concepts and their connections in our database with those in CLICS^16^. We identified some semantic connections that are absent in CLICS. For instance, examples were provided above for senses connected to HAND and WATER, some of which do not appear in CLICS or are not linked to HAND or WATER. Another example is the number of senses linked to HEART, which reaches 80, including <center>, <core>, <spirit >, <emotion>, <courage>, <thought>, <breast>, <something heart-shaped>, <desire>, <the central part of something>, <attention>, <chest>, <feeling>, <mind>, <middle>, <soul>, <encouragement>, <interest>, <stomach>, <will>, <cardiac>, <lover>, <master>, <sympathy>, <change>, <completely>, <nut> etc. In the Cross-Linguistic Database of Polysemous Basic Vocabulary, HEART is related to <spirit>, <soul>, <liver>, <mind>, <stomach>, <chest>, <breath or breathe>, <inside>, <belly>. These semantic connections also appear in CLICS, while remaining connections are not represented in CLICS. In CLICS, <core> is not connected to HEART, but in our database, the connection between <core> and HEART links HEART and EYE together, even though the connection between <core> and EYE is rare in our database. Similarly, <attention> is not linked to HEART in CLICS, but in our database, the connection between <attention> and HEART links HEART and EAR together. The Cross-Linguistic Database of Polysemous Basic Vocabulary also missed linking a few concepts like LUNG, GHOST, KIDNEY, whose connections with HEART are present in CLICS. In the Cross-Linguistic Database of Polysemous Basic Vocabulary, concepts are linked to a more diverse set of senses that are absent in CLICS, while our database misses a small portion of semantic connections present in CLICS.

## Polysemous semantic networks of basic vocabulary across languages

### Building the semantic network

We created semantic maps covering multiple domains. The semantic map is a graph consisting of nodes, representing basic concepts and their related senses, with edges connecting the nodes to symbolize the relationships between the senses and between the senses and concepts. First, the data of polysemous basic vocabulary were converted into a list with the source (concepts) in the first column, the target (senses related to concepts) in the second column, and values indicating the frequency of lexical items expressing a sense (see the supplementary material). If a concept corresponds to multiple synonyms in a language, then the frequency of the extended sense of these synonyms will be weighted by dividing it by the number of synonyms. Second, we visualize the weighted semantic maps with Gephi9^33^, a software solution that allows us (1) to filter out rare patterns of co-expression based on the weight of the edges so as to generate stronger hypotheses about the structure of the semantic field of basic conception and core word, and (2) to detect groups or communities in the map by using the measure known as modularity. Modularity classes are automatically computed using the Louvain algorithm In Gephi^34^. The Louvain algorithm is widely recognized as an efficient optimization method that employs a node-moving strategy to achieve modularity optimization. It often produces communities with a high modularity score, indicating a meaningful and well-defined community structure^35,36^. A community is a cluster of nodes with a high density of connections (i.e., with many connecting edges) within the community and a low density of connections (i.e., with fewer edges) outside the community^37^. In the context of the visualizations discussed in the present study, a community should be thought of as a cluster of senses that are closely linked and as a whole are only weakly linked to other clusters.

**Figure 2:**
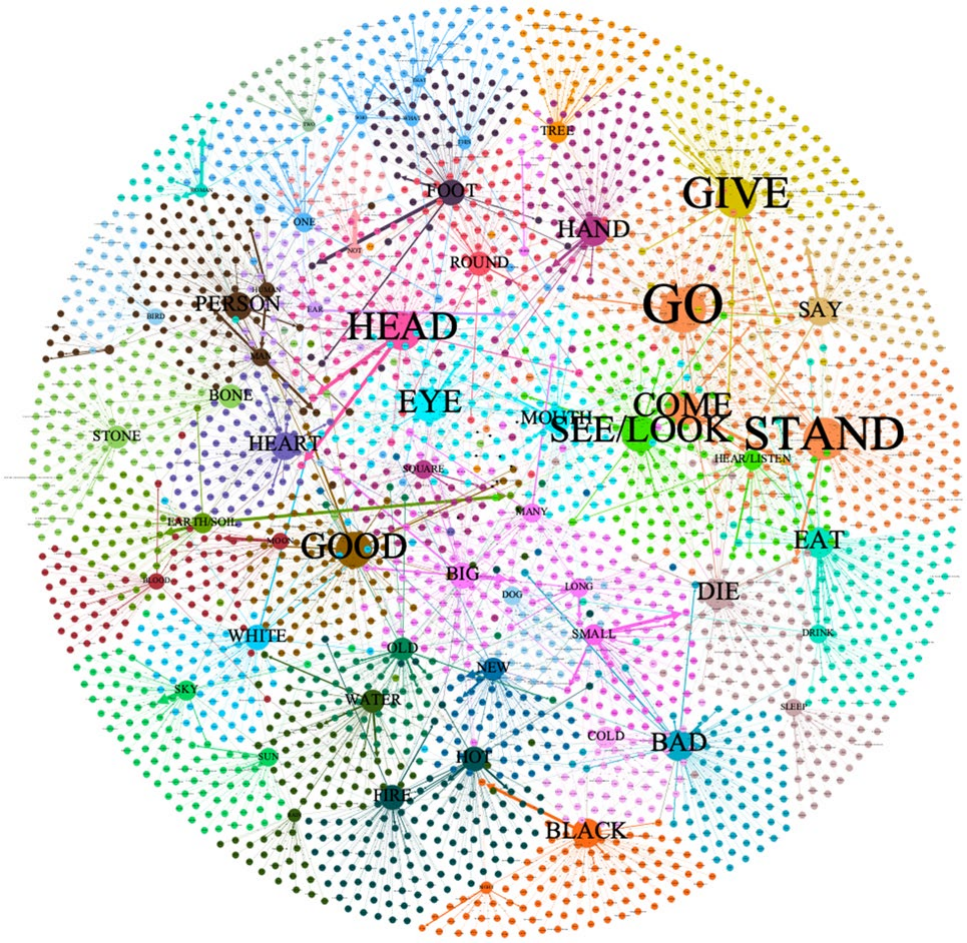
Visualization of a polysemous semantic network using the Fruchterman Reingold algorithm in Gephi (https://gephi.org). The thickness of the edges represents the frequency of occurrence of senses, while the size of the nodes is based on the out-degree of edges connection to nodes. The colors of nodes are based on modularity classes. Modularity analysis is used in order to automatically identify communities, namely clusters of nodes that are more tightly connected to each other. Thirty-four clusters are identified in the graph by different colours, such as orange (GO, COME, STAND), silver pink (DIE, SLEEP) light-blue (EYE, MOUTH), violet (MANY, BIG, LONG, SMALL).

The Fig. 2 provides a visualization of both the relationships between concepts and senses as well as their frequencies, represented by the thickness of the edges connecting the nodes. In total, the semantic map comprises 2941 nodes (i.e., concepts and senses) connected by 3573 edges. The weight of the edges reflects the frequency of lexical items expressing a given sense, with thicker edges indicating higher frequencies. Due to the complex structures and connections in semantic maps, and as we will specifically focus on the structure of this map, we will refer to the map as a “Semantic Network” in the following sections. The network exhibits the broad topological structure of polysemes observed in our data, and modularity analysis reveals the presence of 34 communities of basic concepts (see the supplementary material). These communities are indicated by different colors on the nodes and reflect groups of concepts that are mediated by frequent senses.

To gain a rapid understanding of a constructed semantic network, an effective method can be employed. Evaluating the significance of a node involves utilizing the PageRank algorithm^37^, which takes into account both the quantity and quality of incoming links. Nodes with higher connectivity probabilities play more significant roles in the network. PageRank, a component of the Google search engine, has been effectively utilized for predicting the centrality of concepts in semantic data^20^. Both human memory and internet search engines confront a common computational challenge: identifying those items that are relevant to a query from a large network of interconnected pieces of information. Computing PageRank for a semantic network can predict which items are particularly prominent in human memory^20^. In a fluency task focus on human responses, people are shown a letter of the alphabet and asked to name the first word beginning with that letter that came to mind. Creating a network from word-association norms involves linking words used as cues in the association task to the words named as their associates. The indegree of a node reflects the number of words for which the corresponding word is generated as an associate. Computing PageRank for a network takes into account that the cues themselves differ in their prominence in memory. The PageRank outperforms two measures of the prominence of words in memory: word frequency and associate frequency. The PageRank of a word provides a new way to predict the prominence of items in memory from word-association data when designing or modeling memory experiments^20^.

Similarly, our polysemous semantic networks also exhibit a structure similar to the network created from word-association norms. In polysemous semantic networks, the concepts GO (0.018), GIVE (0.017), STAND (0.016), HEAD (0.015), and GOOD (0.015) are most likely to receive links. The numbers in brackets represent the probability that each concept is randomly linked to. Meanwhile, YOU (0.001), I (0.001), THREE (0.001), WHO (0.002), and THAT (0.003) have the lowest probability of receiving links. The number of senses involved in the concept strongly affects the probability of nodes being linked. Utilizing the PageRank is possible to quantify the importance of a concept or a sense in the semantic network in a simple way.

Considering that concepts are linked to a more extensive set of senses, the polysemous semantic network created from the Cross-Linguistic Database of Polysemous Basic Vocabulary is dense. The database primarily focuses on a specific set of fundamental concepts, serving as pivots in the semantic network, as explained in the terminology definition. This implies that this network does not have the ability to expand indefinitely, compared to Colexification networks generated from CLICS^16,38^, which focuses on large-scale concepts. Without introducing new concepts, the overall structure of the polysemous semantic network is unlikely to change. If more languages were included, the probability of widening edges or increasing nodes with a single edge in this polysemous network would be greater than the probability of increasing nodes with multiple edges, resulting in a denser network.

What are the more characteristics of the basic concepts network? We will now observe how the semantic network is organized through links within semantic domains and across semantic domains respectively.

#### Intra-domain semantic connections

**Figure 3:**
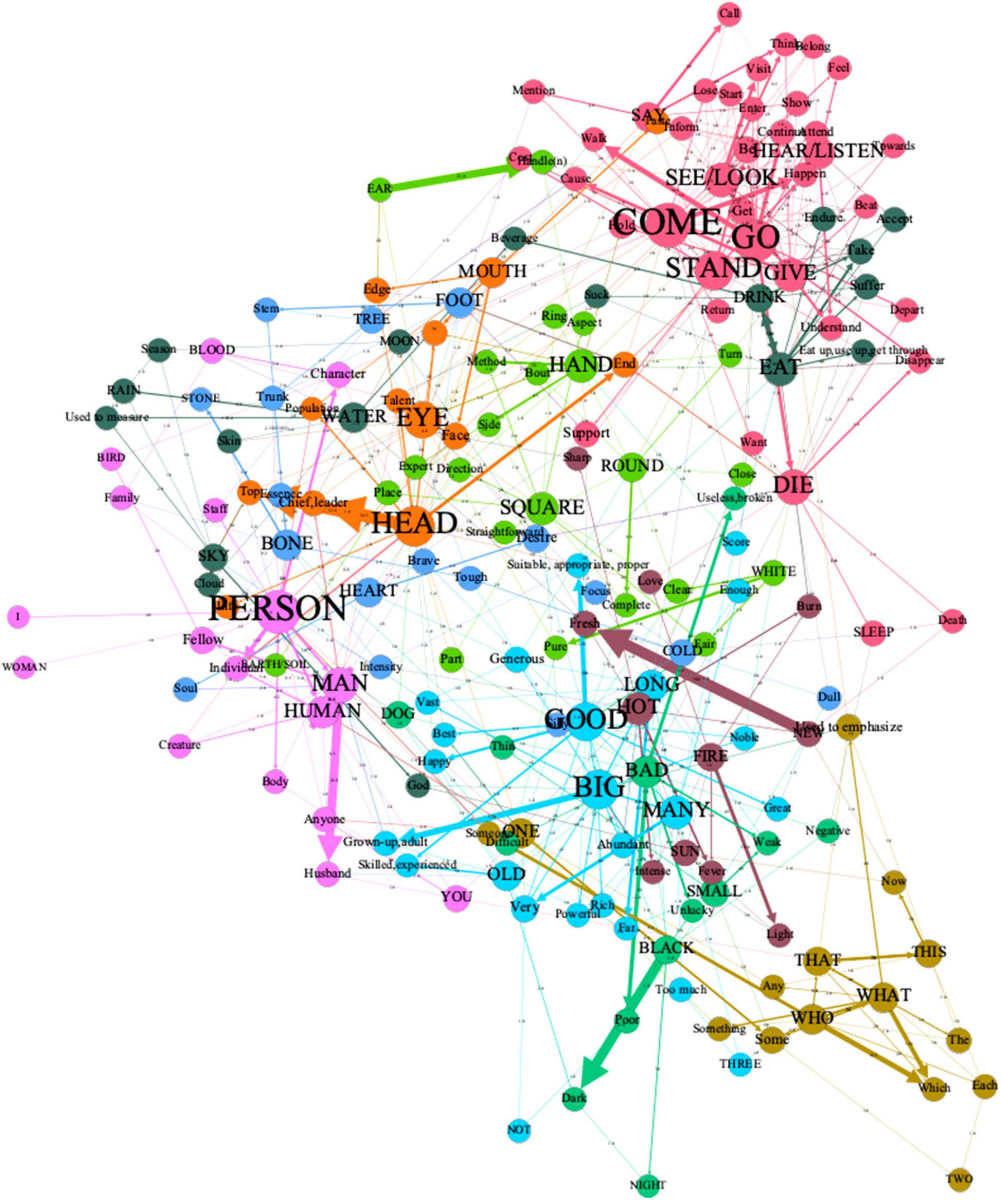
Visualization of a polysemous semantic network using the Yifan Hu algorithm in Gephi (https://gephi.org). It has been filtered out the nodes that are supported by only one and two senses in the whole sample. The modularity analysis identifies five clusters, respectively around GO-GIVE, HEAD-EYE, PEERSON-MAN, BIG-GOOD, and WHAT-WHO.

There exist rare polysemes that occur only once in our dataset connecting the cluster. We prune the least informative edges, that is, the edges with the lowest weight, to highlight those frequent relations. Having filtered out the nodes that are supported by only one and two senses in the whole sample, we end up with the graph of Fig. 3. Filtering was performed using the ‘Filters’ menu, specifically within the ‘Topology’ section, utilizing the ‘Degree range’ option. Based on Fig. 3, we can see that the 60 concepts are clustered into 10 communities (see the supplementary material), and the graph comprises four main subgraphs. They are the semantic field of Motion (COME, GO, STAND, GIVE, SEE/LOOK, HEAR/LISTEN, DIE, DRINK, EAT, SLEEP) and associated senses, the semantic field of body parts (HEAD, EYE, MOUTH, HAND, EAR) and associated senses, the semantic field of human entities (BIRD, MAN, PERSON, YOU, I, HUMAN, WOMAN), and the semantic field of quality and physical attributes (BIG, MANY, LONG, GOOD, OLD, NOT, THREE). In summary, the modularity analysis identifies five clusters, respectively around GO-GIVE, HEAD-EYE, PEERSON-MAN, BIG-GOOD, and WHAT-WHO.

It shows, as already noted by Georgakopoulos et al., that the field of mental perception mediates between vision and hearing^11^. The cognition sense <understand>, <pay attention to>, <accept>, <bear>, <belong>, <call/call on>, <consult>, <feel>, <get>, <taste>, <try/try do>, mediate between the domains of SEE/LOOK and HEAR/LISTEN. The heavy-weight edge is between <understand>, <pay attention to> and SEE/LOOK, HEAR/LISTEN. Some semantic fields of SEE/LOOK and HEAR/LISTEN converge with the information obtained from CLICS database but some of which are new, such as <accept> of both, <judge>, <diagnose>, <appear> of SEE/LOOK^11^. Additionally, the different weights of connection suggest that sight has more frequent extended paths than hearing.

GO, COME and STAND are clustered as a community in both Figs. 2 and 3. COME and STAND share senses: <appear, produce>, <arrival>, and <cost>; GO and STAND share senses <become>, <continue>, <depart>, <get>, <join>, <support>, <take>; COME and GO share <develop>, <end>, <fit>, <lead>, <return>, <set out>, <turn out>, <visit>, <walk>. They all share <attend>, <be>, <endure>, <enter>, <happen>, <lose>, <start>. Although STAND and DIE frequently mean <stop>, DIE is not clustered with them.

There is a strong bond between EAT and DRINK. EAT and DRINK share these senses: <smoke>, <take>, <suck>, <suffer>, <absorb>, <swallow>, <accept>, <corrosion>, <cost, consume>, <embezzle>, <exploit>, <failure>, <listen/listen attentively>. <cock> and <fellow> make a subtle connection between DOG and BIRD. <opening>, <edge>, <face>, <wound> mediate EYE and MOUTH. Senses <day>, <god>, <weather> mediate SKY and SUN. Across these diverse communities, we illustrate senses that have been less appreciated heretofore.

Some unique semantic relationships are also discovered in the semantic network. WOMEN are not as closer to PERSON and HUMAN as MEN. The word MAN is often associated with multiple senses and has been assigned the concepts of HUMAN and PERSON in various languages (e.g., Armenian, Basque, Bengali, German, Tamil).

And it's important to pay close attention to the relationship between opposite pairs in a semantic network. Most opposite pairs referred to, such as GOOD/BAD, BIG/SMALL, NEW/OLD, and WHITE/BLACK are distant from each other in the semantic network and do not share any common senses. The opposite pair COLD/HOT is only connected through the sense of <temperature>. One principle that might explain why opposite pairs are not close in the semantic network is the Goldilocks principle of colexification, as proposed by Brochhagen and Boleda^39^. According to this principle, meanings colexify if they are neither too unrelated nor too closely related but are just right. Meanings that are often alternatives in context, particularly those expressing opposites, are less likely to colexify than other kinds of relations. Meanings that stand in opposition to one another are less likely to be expressed by the same form than those standing in part-whole or subsumption relations. One force is the cognitive pressure for simplicity, which increases the colexification rate for opposites. The other competing force—the need for languages to be informative in supporting accurate information transfer—drives languages toward complexity, pulling the colexification rate for opposites down^39^. This implies that opposite concepts, often alternatives in the same context, will not be connected to common senses. As a result, members of opposite pairs are identified as different communities. There are quite a few senses that can frequently link to these common concepts, such as GOOD, BIG, and OLD. In Fig. 3, these concepts are identified as a community due to their frequent connections with positive senses. Conversely, BAD, SMALL, and BLACK are identified as another community because they are more closely associated with negative senses.

#### Inter-domain semantic connections

Beyond intra-domain semantic connections, we are more interested in inter-domain semantic connections, i.e. mapping from one domain to another. Polysemy networks help us discover there are number of extended patterns that across semantic domains in Fig. 2. (1) STONE and BONE are related to <hard kernels> 4 times and 3 times, respectively, in the database. Both STONE and BONE both are also related to the sense of <seed> and <pit>. Therefore, STONE and BONE are clustered as a community. (2) <Emmenia> mediates between BLOOD and MOON. (3) HAND and SQUARE are linked by senses <direction>, <method>, <side>. (4) FIRE and HOT are bonded by <anger>, <burn>, <fever>, <heat>, <intense>, <jealousy>, and <sun>. (5) NIGHT and BLACK are tied together by <dark>. (6) YOU, THIS, THAT, WHO, WHAT, ONE, these pronouns belong to different domains but serve similar referential functions, known as deixis. Some of them are synonymous and share senses such as <any>, <anyone>, <anything>, <each>, <some>, and <something>. (7) MANY, BIG, LONG, and THREE are clustered as a community. MANY and BIG share senses <abundant>, <enough>, and <heavy>; LONG and BIG share senses <deep> and <generous>; MANY, THREE, and BIG are all related to the sense <too much>.

Figure 3, in which we keep only the senses connected by edges of weight 3 and more, clearly shows that there are several solid articulation points between the subnetworks motion, body part, and physical attributes. For example: <End> mediates between the concepts of DIE, COME, GO, HEAD, and FOOT. <Handle (N)> mediates between EAR, HAND, and SEE/LOOK. <Handle (V)> mediates HAND and GO. <Taste> mediates between HEAR/LISTEN, SEE/LOOK, and MOUTH. <Turn> mediates ROUND, GIVE, and HAND. <Chief, leader>, <individual>, and <talent> mediate MAN and HEAD. HUMAN, PERSON, and HEAD are all related to ONE. <Turn> mediates ROUND, GIVE, and HAND. <Grown-up, adult> mediates BIG, OLD, MAN, HUMAN. <Close> mediates HOT, ROUND, and GO. <Useless, broken> mediates BAD, COME, and DIE.

In terms of modularity, the GO-GIVE community is associated with the PERSON-MAN cluster through the HEAD-EYE. Our semantic networks show that inter-domains connections between motion, human entities, and pronouns are mediated by body parts and physical attributes connections, i.e., via the domains of embodied cognition.

### Embodied relations are embodied in the polysemy network

How are the events and objects represented and organized in the mind? Embodied theories propose that our cognition is grounded in our bodily experiences and interactions with the world. A basic claim of the embodiment framework is that all psychological processes are influenced by body morphology, sensory systems, motor systems, and emotions^40^. As such, several semantic domains, such as body parts, motions, physical attributes, quality, and quantity, are considered to play important roles in embodied cognition.

“Peculiar nature of our bodies shapes our very possibilities for conceptualization and categorization”^41^. The body plays a central role in embodied theories, as it is the primary vehicle through which we interact with the world. Our bodily sensations, such as touch, proprioception (our sense of where our body is in space), and visceral sensations, are thought to shape our mental representations of concepts and objects. As the human body serves as a primary source domain for languages to conceptualize the world, it would be worth wondering what is the position of body parts in the semantic network of basic concepts, and what is their relationship with other basic concepts. Based on Fig. 3, the nine body concepts belong to three different communities: HEAD, MOUTH, EYE; HEART, FOOT, BONE; EAR, HAND. The concept of body parts belongs to different communities, meanwhile, they have rich extensions, which both result in a wide range of body fields in the semantic network. In the previous section, we noted that the connections between motion, human entities, and pronouns are often mediated by connections to body parts. Representations of various aspects of our bodies (e.g., their configural structure and layout in space) thus play critical roles in perception and skilled action^9^.

BLOOD does not belong to any of the three communities. BLOOD is different from other body parts because it relates to fewer senses than other body parts. In Estonian figurative descriptions, there is a continuum of exploitable body parts, internal and external, and more diversely described emotions. In contrast to the internal body parts and fluids such as the heart, blood, and nerves, the external and movable parts of the body such as the head and its subparts (the eyes, the mouth, the nose) and hands are more heavily exploited for the purpose of the emotion expression^42^. This trend is also reflected in our cross-linguistic database, which shows that BLOOD relates the fewest senses compared to other body parts, indicating that it has the weakest ability for semantic change.

Physical attributes, quality, and quantity have a similarly broad domain as body parts, but their modularization is complex and intertwined. The communities include physical attributes, quality, and quantity: BIG, MANY, LONG, GOOD, OLD, NOT, THREE; HOT, NEW, SUN, FIRE; WHITE, ROUND, SQUARE, HAND, EAR, EARTH/SOIL; BAD, BLACK, SMALL, NIGHT, DOG. They belong to different semantic domains making them less homogeneous than body parts. They are only observed together because they are all adjectives in most languages. Adjectives are different from nouns and verbs in mental representation. For nouns and motion verbs, the literal and metonymic senses of polysemous words are semantically related, share similar contexts, and can be stored in a unified lexical representation in the mental lexicon. Their metaphorical senses are perceived as unrelated, share fewer contexts, and are more likely to have distinct representations. In contrast, with adjectives, metonymic senses significantly overlap with literal senses on the one hand and metaphorical senses on the other. As a result, people may struggle more with adjective classification than with other word classes. The more similar the senses are, the harder it is to distinguish them. The differences between adjectives, nouns, and verbs may be explained by the fact that adjectives are much less concrete and imageable than nouns or verbs^43^.

The embodied cognition hypothesis suggests that cognition is mediated by representations expressed in the vocabulary and format of sensory and motor representations^42^. Motion words are closely linked to body parts in the semantic networks, they are processes that the body’s interactions with the world. Motion, like body parts and attributes, has a rich set of senses. However, in contrast to body parts and attributes, the concept of motion is highly concentrated and clustered within a community and does not occupy a large field in the semantic network, the probable reason is that motions are more concrete than physical attributes. Verbs denote actions and typically have multiple arguments, which help us to imagine the situation being described by the verb more vividly^43^. Verbs such as EAT and DRINK do not cluster with other motion verbs because they have different argument structures.

Natural entities neither form a cohesive community nor are they the most prominent in their respective communities. Instead, they are mixed in communities based on body parts, physical properties, and motions. The number of senses linked by natural entities is far less than that of motions, body parts, and physical attributes. In a semantic network, concepts such as body parts, actions, and physical attributes are more salient in terms of their node prominence and pivot function compared to natural entities. Conceptual knowledge is built through experiencing the environment, where elementary perceptual attributes form the basis of concept representations that are built incrementally^44^. Natural entities cannot exist independently of our field of experience, and therefore, cannot be easily clustered into a close-knit community. Embodied cognition theorists aim to explain the full range of perceptual, cognitive, and motor capacities we possess as capacities that are constitutively dependent upon aspects of an agent’s body^45^. Compared with an agent's body, body motions, and features conceptualized through human experience, natural entities are objects and belong to the world outside the subject. Therefore, natural entities have fewer extensions, and their node size is not prominent across various communities.

Because of their limited number of members and restricted ability to generate extended meanings, human entities, and pronouns have less prominence in a semantic network compared to motions, body parts, and physical attributes. Despite their close intra-domains connections, the semantic fields PERSON-HUMAN and WHO-WHAT do not play a pivotal role in linking other fields. The WHO-WHAT semantic domain is also the least prone to polysemy across all domains, which is related to its function. Therefore, it has the least association with other semantic domains and a close internal relationship, making it the most independent semantic domain of all.

The information obtained from embodied experience can be considered a form of distributional information, which is analogous to the information captured by distributional semantic models^44^. Our proposal is that the polysemous network of basic vocabularies across different languages can serve as another empirical field for the study of embodied cognition.

The existence of a certain sense in a particular language may not be universal, but it can suggest a potential semantic link in human cognition. The conceptual distribution patterns formed by the semantic connections discussed above are not universal but shared, which means that they are present in multiple individual languages or language families, but not necessarily present in all of them. Each specific connecting line should reflect the existence of at least one documented case of a direct lexical connection between these two senses in any of the world's languages^27^. When these concepts are linked together by shared senses, they form a polysemous network across languages that is contributed to by people who speak these languages. However, these semantic connections should be conceived as generally independent of any specific language. The understanding that a sense forms a crucial link between two other senses should not depend on any particular language but rather on the intrinsic properties of each sense. This organization of meaning is not driven by idiosyncrasies of any specific language but by universal characteristics of the real world as perceived by the human mind and filtered through human activities^27^.

Polysemy reveals general cognitive processes^8^, and implies any kind of proximity of corresponding concepts in a mental space^7^. Meanwhile, semantic network implies that certain semantic map models correspond to cognitive reality to some degree^46–48^. Therefore, the polysemous network of basic vocabularies can also be viewed as a form of cognitive network. Baker et al. gained insights into the structure of associative knowledge by investigating difficulties in picture naming among individuals with aphasia. They conceptualized target/produced word pairs as interconnected elements shaped by associative knowledge. Utilizing multiple lexical networks, they depict conceptual associations in the human mind. One layer specifically represents semantic connections. Words are linked in the network if they are synonyms^49^. Distinct from the semantic network created based on word-association norms and target/produced word pairs^20,49^, the polysemous network of basic vocabularies across languages doesn't merely represent a cognitive network but, instead, a shared cognitive network reflecting fundamental human experiences. The significance of body parts, motions, and features closely related to human experience is prominently evident in this network, occupying important spaces or serving as crucial bridges in the semantic network.

## Conclusion

We have created a Cross-Linguistic Database of Polysemous Basic Vocabulary to explore a broad range of senses for and assess the patterns of polysemy across languages. Our study covers 60 basic vocabularies in 61 languages, providing 11,841 senses from 3736 entries. To demonstrate the database's research potential, we present case studies that use automatically generated weighted semantic maps. These maps reveal cross-linguistic semantic structures, comprising 2941 nodes connected by 3573 edges. The significance of body parts, motions, and features closely related to human experience is prominently reflected in this network, occupying important space or serving as crucial bridges in the semantic network. By linking these concepts through shared senses, they form a polysemous network across languages. While the presence of a specific sense in one language may not be universal, it can indicate a potential semantic connection in human cognition. The distribution patterns of these semantic connections are shared semantic connection in human cognition, rather than a universal feature that necessarily exists in every individual's mind. As a result, the polysemous network of basic vocabularies across languages represents a shared cognitive network of fundamental human experiences. In addressing the question of how concepts related to fundamental human experiences are organized within the human mind, insights are gained from this shared cognitive network of fundamental human experiences. Overall, concepts representing body parts, features, and motions, which are closely tied to human experience, occupy extensive areas. Specifically, concepts representing motion in the network have a diverse set of senses, with the concept highly concentrated and clustered within a community. Concepts representing body parts and features play significant roles, serving as crucial bridges across other semantic domains like motion and human entities.

An increasing number of cognitive studies are incorporating semantic connections to provide explanations for more general cognitive phenomena by investigating their correlation with factors such as empirical affective ratings, word-norm associativity (obtained by prompting subjects to produce words in response to a cue), and the similarity of contextual usage^38,39,49^. Although the Cross-Linguistic Database of Polysemous Basic Vocabulary focuses on a select group of core concepts, these concepts are intricately linked to a substantial number of senses. The database offers valuable semantic connections, some of which are missing in other databases^7,16,17^. The Cross-Linguistic Database of Polysemous Basic Vocabulary holds the potential to make a contribution to research aimed at unraveling the nature of cognitive proximity or investigating the determining factors behind such proximity.

### Supplementary Information

Supplementary Information.

## Data Availability

The Cross Linguistic Database of Polysemous Basic Vocabulary is available for open-access and can be accessed both on the website https://github.com/EL-CL/CLD_Polysemous_Basic_Vocabulary and through supplementary materials. The supplementary material contains five sheets. The first sheet is the Cross-Linguistic Database of Polysemous Basic Vocabulary. The second sheet consists of dictionaries and their publication information and dates, which were used to collect word meanings. The third sheet is a converted version of the Cross-Linguistic Database, used as input data for generating the Polysemous Semantic Networks. We have uploaded a script named 'preparing_network_data.py' with the purpose of processing data from the Cross-Linguistic Database of Polysemous Basic Vocabulary for visualization in Gephi. The fourth sheet contains information on the modularity classification of nodes in Fig. 2, indicating that the 60 concepts and their senses belong to 34 different communities. The fifth sheet contains information on the modularity classification of nodes in the filtered network shown in Fig. 3, indicating that the 60 concepts and their senses belong to 10 communities.
